# The Roses Ocean and Human Health Chair: A New Way to Engage the Public in Oceans and Human Health Challenges

**DOI:** 10.3390/ijerph17145078

**Published:** 2020-07-14

**Authors:** Josep Lloret, Rafael Abós-Herràndiz, Sílvia Alemany, Rosario Allué, Joan Bartra, Maria Basagaña, Elisa Berdalet, Mònica Campàs, Arnau Carreño, Montserrat Demestre, Jorge Diogène, Eva Fontdecaba, Mireia Gascon, Sílvia Gómez, Angel Izquierdo, Lluïsa Mas, Montse Marquès, Juan Pedro-Botet, Maria Pery, Francesc Peters, Xavier Pintó, Marta Planas, Ana Sabatés, Joan San, Anna Sanchez-Vidal, Martí Trepat, Cristina Vendrell, Lora E. Fleming

**Affiliations:** 1 SeaHealth Research Group-Institute of Aquatic Ecology & LIPPSO-Dept. of Chemistry, University of Girona, 17003 Girona, Spain; acarre93@gmail.com (A.C.); marta.planas@udg.edu (M.P.); joan.san@udg.edu (J.S.); 2 Department of Primary Health Care, Institut Català de la Salut, Government of Catalonia, 08013 Barcelona and 17480 Roses, Spain; 24025rah@gmail.com (R.A.-H.); efontdecaba.girona.ics@gencat.cat (E.F.); cvendrell.bcn.ics@gencat.cat (C.V.); 3 History Museum of Sant Feliu de Guíxols, Sant Feliu de Guíxols, 17220 Catalonia, Spain; silvia.alemany@guixols.cat; 4 D.G. Fisheries and Maritime Affairs, Government of Catalonia, 08017 Barcelona, Spain; rosario.allue@gencat.cat; 5 Allergy Section, Pneumology Department, Institut Clínic Respiratori (ICR), Hospital Clínic, 08036 Barcelona, Spain; JBARTRA@clinic.cat; 6 Allergology Unit, Hospital Germans Trias i Pujol, 08916 Badalona, Spain; mabato75@hotmail.com; 7 Institut de Ciències del Mar, CSIC, 08003 Barcelona, Spain; berdalet@icm.csic.es (E.B.); montse@icm.csic.es (M.D.); cesc@icm.csic.es (F.P.); anas@icm.csic.es (A.S.); 8 Marine and Continental Waters Programme, IRTA, Sant Carles de la Ràpita, 43540 Catalonia, Spain; monica.campas@irta.cat (M.C.); jorge.diogene@irta.cat (J.D.); 9 ISGlobal (Global Health Institute Barcelona), Barcelona, Spain; mireia.gascon@isglobal.org; 10 Department of Experimental and Health Sciences, Universitat Pompeu Fabra (UPF), Barcelona, Spain; 11 CIBER Epidemiología y Salud Pública (CIBERESP), Madrid, Spain; 12 Departament of Social and Cultural Anthropology, Autonomous University of Barcelona, Bellaterra (Cerdanyola del Vallès), 08193 Catalonia, Spain; silvi.gomezmestres@gmail.com; 13 Medical Oncology Service, Catalan Institute of Oncology, Hospital Universitari de Girona Doctor Josep Trueta, 17007 Girona, Spain; aizquierdo@iconcologia.net; 14 Sub-direcció Regional a Girona, Catalan Public Health Agency, Government of Catalonia, 17002 Girona, Spain; lluisa.mas@gencat.cat (L.M.); mtrepatq@gencat.cat (M.T.); 15 Laboratory of Toxicology and Environmental Health, School of Medicine, IISPV, Universitat Rovira i Virgili, 43201 Reus, Spain; montserrat.marques@urv.cat; 16 Department of Medicine, Hospital del Mar & Universitat Autònoma de Barcelona, 08003 Barcelona, Spain; JPedrobotet@parcdesalutmar.cat; 17 Servei d’Espais Naturals Protegits, D.G. Environmental Policies and Environment, Government of Catalonia, 08036 Barcelona, Spain; maria.pery@gencat.cat; 18 Unitat de Lípids i Risc Vascular, Servei de Medicina Interna, Hospital Universitari de Bellvitge, Idibell, University of Barcelona, CiberObn, 08907 L’Hospitalet de Llobregat, Spain; xpinto@bellvitgehospital.cat; 19 Department of Earth and Ocean Dynamics, University of Barcelona, 08028 Barcelona, Spain; anna.sanchez@ub.edu; 20 European Centre for Environment and Human Health, University of Exeter Medical School, Cornwall TR1 3HD, UK; l.e.fleming@exeter.ac.uk

**Keywords:** marine conservation, public health, participatory process, citizen science

## Abstract

Involving and engaging stakeholders is crucial for studying and managing the complex interactions between marine ecosystems and human health and wellbeing. The Oceans and Human Health Chair was founded in the town of Roses (Catalonia, Spain, NW Mediterranean) in 2018, the fruit of a regional partnership between various stakeholders, and for the purpose of leading the way to better health and wellbeing through ocean research and conservation. The Chair is located in an area of the Mediterranean with a notable fishing, tourist, and seafaring tradition and is close to a marine reserve, providing the opportunity to observe diverse environmental conditions and coastal and maritime activities. The Chair is a case study demonstrating that local, collaborative, transdisciplinary, trans-sector, and bottom-up approaches offer tremendous opportunities for engaging coastal communities to help support long-lasting solutions that benefit everyone, and especially those living by the sea or making their living from the goods and services provided by the sea. Furthermore, the Chair has successfully integrated most of its experts in oceans and human health from the most prestigious institutions in Catalonia. The Chair focuses on three main topics identified by local stakeholders: Fish and Health; Leisure, Health, and Wellbeing; and Medicines from the Sea. Led by stakeholder engagement, the Chair can serve as a novel approach within the oceans and human health field of study to tackle a variety of environmental and public health challenges related to both communicable and non-communicable diseases, within the context of sociocultural issues. Drawing on the example provided by the Chair, four principles are established to encourage improved participatory processes in the oceans and human health field: bottom-up, “think local”, transdisciplinary and trans-sectorial, and “balance the many voices”.

## 1. Introduction

Involving and engaging diverse publics are crucial for effective prioritization and the dissemination and implementation of research about the complex interactions between environments and health [1,2]. Researchers increasingly seek to engage communities, patients, non-governmental organizations (NGOs), and other stakeholders as partners in environmental and public health research and management [3,4,5,6,7,8]. The importance of stakeholder–researcher–decision-maker interactions in co-producing knowledge that can be actionable and sustainable in decision-making has been demonstrated [9,10,11]. Furthermore, institutions and funders increasingly require tangible public involvement not only in research, but also in the implementation and practice of its outcomes [2]. The emergence of complex socio-environmental challenges, such as climate change adaptation, sustainable development, and disaster risk reduction, as well as complex public health issues such as obesity and mental health disorders, has coincided with calls for more integrative and participatory approaches to scientific research. To this effect, co-production (or participatory action research) aims to contribute by addressing these societal challenges through integrating stakeholders in all phases of research, from design to execution [11].

However, how co-production actually occurs in practice is less clear [11,12]. One way to foster meaningful knowledge co-production is through transdisciplinary and trans-sector approaches, which are integrative and participatory processes that bring together diverse academic and non-academic actors, disciplines, and knowledge bases [9,13]. This new holistic approach allows for an integrated view with completely different outcomes from what one would expect from just the addition of the parts [14].

In environmental sciences, placing stakeholders at the center of the development and implementation of the decision-making process is increasingly seen as an effective approach to reduce the risk of failing to implement sustainable environmental management strategies [15]. A similar trend has occurred in public health science, with the promotion of individual and community involvement in decisions affecting people’s health and leading to self-reliance [16]. While a quarter of the global burden of disease can be prevented through known strategies to manage environmental health risks [17], a significant portion of the creation of health and wellbeing lies outside the health care sector [16]. The World Health Organization (WHO) recognizes that the health of populations can be improved by reducing the exposure to health risks posed by different environmental factors; therefore, there is a need to preserve the health of natural environments, raise awareness, and catalyze the development of multi-sectoral policies and programs to reduce exposure to these environmental risks [18]. Furthermore, since natural environments provide a space for citizens to enjoy outdoor activities that can be beneficial for their health and wellbeing [19,20,21], initiatives, such as “green prescriptions”, which involve people undertaking activities in natural environments that benefit their health and wellbeing, with subsequent population-wide impacts, have been fostered [22].

Several “top-down approaches” without the involvement of user groups have been proposed to integrate science into the policy process in marine ecosystems and public health [23]. However, these top-town initiatives have often struggled to make an impact on many of the complex environmental and health challenges facing us today. A classic example is fisheries management. For years, fishery policies have been formulated at global and national levels using a macro approach to increasing food availability, but they have frequently failed to prevent the over-exploitation of fish populations, or they have even exacerbated the problems through mismanagement [24]. In recent decades, “fisheries co-management” (defined as a collaborative and participatory process of regulatory decision-making among representatives of local fishers, governments, NGOs, and fisheries scientists) has become more popular [24].

In public health sciences, national level campaigns and nationally standardized programs have likewise often struggled to make an effective impact on health issues [16,25,26]. It is increasingly recognized that many of the solutions to improve the public health and wellbeing of citizens fall far beyond the scope of international and national health systems, and that local initiatives can contribute to improving health and wellbeing by playing a leading role in local partnership networking [25,26,27]. However, few initiatives exist for public health at the local and regional levels [28,29].

Within the field of oceans and human health (OHH), a relatively new transdisciplinary field of study concerned with the substantive relationships that exist between marine ecosystems and human health and wellbeing [30], several initiatives to foster public involvement, engagement, and participation have recently emerged at both the national and international levels. Some such examples are: The Centre for Environment and Human Health at the University of Exeter in the UK [2]; the OHH Initiative and the harmful algal bloom (HAB) actions, both from the National Oceanic and Atmospheric Administration (US) [31]; and the European projects SOPHIE [14], and OstreoRisk [32]. However, insufficient attention has been paid to local initiatives, which remain relatively unexamined.

The theoretical understanding and practical experience of the OHH Chair (the Chair) in Roses (Catalonia, Spain) are used to explore the challenges and opportunities involved in creating a flexible knowledge space to facilitate local stakeholders’ effective and sustained involvement, engagement, and participation. Using the Chair as a case study, this article aimed to examine the added value brought by local approaches to the research and management of OHH challenges.

Throughout this paper, stakeholders refers to academic experts (i.e., those involved in conducting scientific research); non-academic experts (i.e., professionals who have expertise related to an issue under study, e.g., medical practitioners working in primary health care centers and public hospitals); public health and marine affairs managers (including those working in the regional administration in charge of public health and marine policies); businesses (such as fishers and maritime recreational organizations); and the wider publics (i.e., practitioners, environmental NGOs, patients associations, and environmental educators). Regarding the participatory process, the definitions used by the National Institute for Health Research [33] of “involvement” (the active involvement of the public in projects or organizations), “engagement” (the provision of information and knowledge about research, i.e., scientific dissemination), and “participation” (where people are recruited to take part in research, including trials, focus groups, and questionnaire completion) were followed.

## 2. The OHH Chair: Setting the Scene

**Figure 1:**
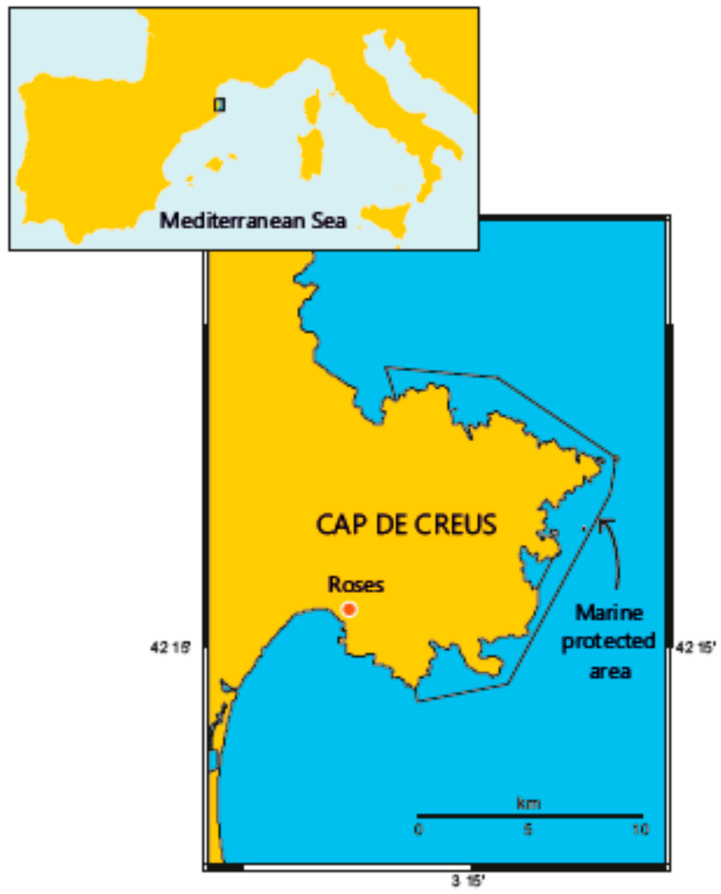
Map of the area where the Oceans and Human Health (OHH) Chair is implemented in the northwestern Mediterranean, showing the City of Roses and the marine protected area of Cap de Creus.

The Chair was founded in 2018, the fruit of the collaboration between the University of Girona, the town of Roses, the Fisher’s Association of Roses, and the Fishmongers’ Guild of Catalonia. It was established in Roses, a town of around 20,000 inhabitants located in the north-easternmost corner of the Iberian Peninsula (Catalonia, Spain; Figure 1), which has an established fishing activity and tourist tradition. The whole area constitutes a “living laboratory” for testing sustained public engagement, involvement, and participation activities. Although most studies related to OHH deal with the “ocean” or the “sea” as a unique factor [14], the complexity and diversity of the stakeholders, coastal and maritime activities, and environmental conditions in the area make it a mosaic of situations to test different hypotheses that have been poorly addressed so far within the OHH paradigm.

**Figure 2:**
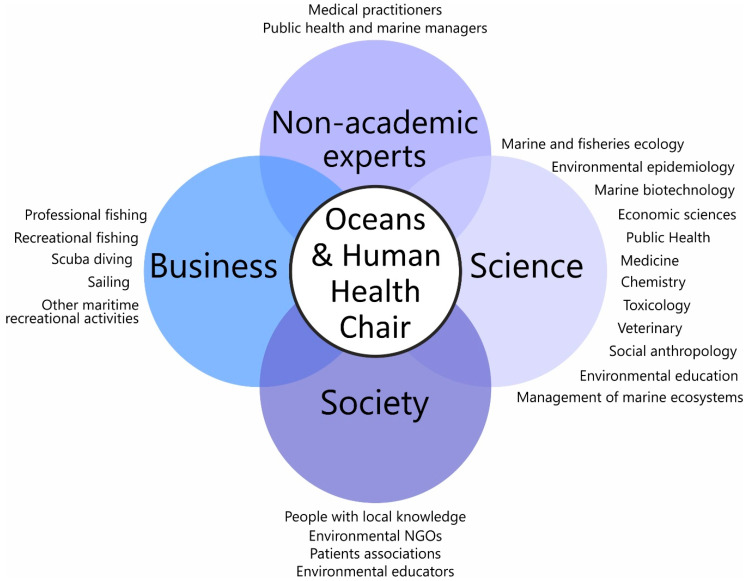
The fundamentals of the Oceans and Human Health Chair in Roses.

The Chair is the first to date to focus on the topic of OHH in Catalonia, Spain, and Europe. It not only contributes to a better understanding of the links between marine ecosystems and human health and wellbeing and involves citizens in a research agendas, but it also shares the generated knowledge about a relatively little-known topic—how protecting and preserving marine ecosystems can also protect, preserve, and improve the health and wellbeing of people—with society at large. The Chair addresses different closely related aspects, such as the research and conservation of marine ecosystems and public health [30,34,35]. Since this purpose can only be achieved if marine ecosystems are considered from a holistic perspective that includes a broad range of academic disciplines [30], the Chair was set up with a transdisciplinary spirit, including marine and fisheries ecology, public health and medicine (including oncology, allergies, cardiovascular risk, and mental health), environmental epidemiology, veterinary medicine (including parasitology), the management of marine ecosystems, social anthropology, chemistry and toxicology, marine biotechnology, environmental education, and economic sciences (Figure 2). The Chair also undertook a trans-sectoral spirit, including government, business, NGOs, education, and science.

## 3. The Role of the Marine Protected Area of Cap de Creus

The town of Roses is located close to the marine protected area (MPA) of Cap de Creus (Figure 1). MPAs are not only a tool used to meet management objectives for the marine environment, but they can also be used in the OHH area as a perfect setting to assess the trade-offs between the conservation of marine biodiversity and the development of human activities that can promote citizen health and wellbeing. An example of such a trade-off is the balance between the environmental risks from unsustainable fishing practices vs. the health benefits derived from the fishery products [36]

First, the MPA of Cap de Creus is an environment where diverse leisure and fishing activities are being developed, and where there is a broad diversity of habitats and species.

Second, the better preservation of the marine environment in the MPA compared with the adjacent non-protected area [37] provides the opportunity to test how habitat protection and quality could be factors that affect the potential health benefits of the marine environment, i.e., to test the hypothesis that the best preserved/high quality marine environments could yield better human health and wellbeing outcomes.

Third, the area offers the possibility of comparing the health and wellbeing benefits of relatively environmentally friendly activities, such as swimming, scuba diving, sailing, and kayaking, with those of other activities that have much greater impacts on the marine environment, such as recreational fishing [38] and leisure boating [39].

Fourth, diverse user behaviors co-exist in the area (e.g., scuba divers diving in large groups vs. those diving in small groups), offering the possibility to assess the influence of these different behaviors on visitor experience and perceptions, which may finally modulate the benefits of these activities for health and wellbeing.

Last, the diversity of users can contribute to the development of citizen science or citizen-led initiatives (understood as scientific research that relies on the active involvement of non-specialized publics), bringing together users, the fishing and tourism industries, and experts to help create awareness of OHH issues and to consider local ecological knowledge (i.e., people’s knowledge about their local environment; [40] and self-reported health (i.e., people’s perception of their health and wellbeing; [41] as potential data inputs for studies.

## 4. Materials and Methods

Stakeholder involvement was sought before, during, and after the creation of the Chair through the dedicated building of relationships and trust over time and following a participatory research action (PAR) approach [42]. This method is based on reflection, data collection, and action that aims to improve health and reduce health inequities through involving the people who, in turn, take actions to improve their own health. The PAR method uncovers the social, environmental, and health aspects that are important benchmarks to ensure equitable access to health by involving stakeholders as participants from the onset.

**Figure 3:**
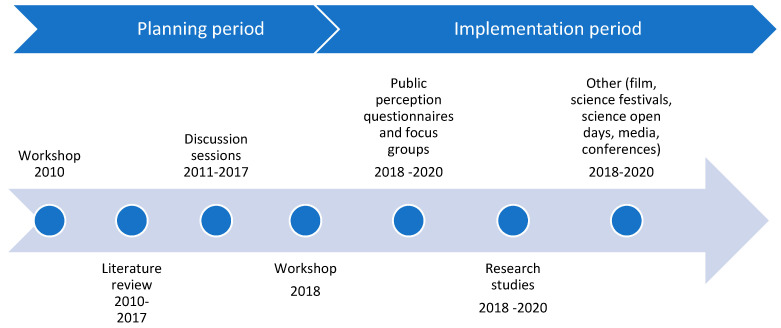
General workflow of the design and development of stakeholder involvement, engagement, and participation.

The design and development of stakeholder involvement, engagement, and participation followed two steps (Figure 3): a planning period (before 2018) and an implementation period (after 2018, when the Chair was founded). During the planning period, the most important challenges facing the OHH field of study were discussed with the stakeholders (all stakeholders were involved in formulating the set of challenges). The involvement of stakeholders during the planning period contributed decisively to prioritizing the research actions, fixing the Chair’s global approach to marine ecosystems and human health. It was particularly important to stakeholders to emphasize the context of the developed Mediterranean coastal countries, exploring the challenges, opportunities, and research gaps related to emerging OHH issues, and highlighting the crucial role of collaboration between different stakeholders.

When the Chair was founded in 2018, stakeholder involvement was officially embedded in its structure through the creation of a Follow-up Committee and an Advisory Board. Stakeholder involvement subsequently continued through an annual meeting with the Follow-up Committee and the Advisory Board. From the discussions held during these annual meetings, minutes were written and an annual report of the activities, along with the agreements about the proposal of new ideas, was elaborated.

During both periods (planning and implementation), the Chair employed a comprehensive array of strategies to construct effective involvement, engagement, and participatory activities in environmental and public health sciences, provided in [15,25,43]. The involvement in activities considered the iterative ongoing setting of priorities with the stakeholders. The public engagement and participation activities were diverse (see Supplementary Table S1 for details) and comprised science open days, science festivals, conferences, and projects in which citizens collaborated with the Chair’s scientists through citizen science approaches. During these activities, the information was collected through different channels: (i) discussion sessions during individual and/or group meetings and workshops; (ii) public perception questionnaires and focus groups; and (iii) feedback from the stakeholders through different means (by email, phone, social media, and face-to-face conversations). Furthermore, existing literature specific to the research field, websites of topic-related projects, and policy documents on human health and environmental issues was collected. The data resulting from the participatory process were analyzed by the academic and non-academic experts (as defined in Section 1).

(i) Discussion sessions

More than 50 people were involved from among the different stakeholder groups, which included the academic scientific community, policy makers, commercial companies (e.g., scuba diving and kayaking clubs and fishing associations), and members of patient and environmental associations, through more than 40 face-to-face individual and/or group meetings and two major workshops held in 2010 and 2018, coordinated by the University of Girona [44,45]. Discussion sessions (with individuals and/or groups of experts and during workshops) were carried out. Notes were taken and the workshops were recorded for the purpose of capturing the discussions and the proposed ideas. In these sessions, participants were asked to discuss the role of each stakeholder in the Chair and the relationships between stakeholders, as well as to propose participatory ideas. In these sessions, the director of the Chair acted as an observer/facilitator in the sessions, taking observation notes and suggestions that arose. The outputs of the sessions were analyzed by means of the reporting of the opinions and suggestions. The names of the participants were not disclosed for privacy reasons.

The stakeholders belonged to different institutions, mostly in the region of Catalonia (>90% of the stakeholders), and covered the different fields linked to OHH cited in the previous section. To this end, the Chair was also able to set up a local discussion forum on the topic of OHH among the various stakeholders (Figure 2). Because this was a public involvement project in which people were acting as special advisors and activity constituted consultation, collaboration, and co-production of the research, as opposed to data gathering, these activities did not require review by a research ethics committee [33].

(ii) Public perception questionnaires and focus groups

Open and closed questionnaires and focus groups were carried out in the frame of different research projects to gather the opinions and knowledge of different stakeholders, including sea users (scuba divers, sailors, kayakers, etc.), businesses, and oncology patients. These activities required review and approval by the research ethics committee of the University of Girona. Closed-ended questionnaires were analyzed though a general and descriptive overview of answers, whereas open-ended questionnaires were analyzed qualitatively by coding analysis of the answers. The analyses of the focus group involved a transcript of the discussion and a summary of the conclusions drawn.

(iii) Other feedback from the stakeholders

Informal feedback from stakeholders was gathered during the participation and engagement activities through different means in which the stakeholders approached the Chair’s director: by email, phone, social media, and face-to-face conversations (notes were taken without disclosing the names of the persons). These activities did not require formal research ethics committee approval. The outputs were analyzed by means of the reporting of the opinions and suggestions.

(iv) Review of scientific and existing policy documents

The emerging OHH issues were explored by the academic experts of the Chair by means of a literature review on a worldwide scale, but particularly focused on the Mediterranean. It must be noticed that the absence of other stakeholders in this review is in contrast to the bottom-up premise of the participatory approaches of the rest of Chair activities and can introduce some bias. The following resources were used: peer-reviewed journals and books specific to the research field included in scientific databases (e.g., Science Direct, PubMed, Web of Science) and gray literature (e.g., papers, reports, technical notes, and other documents produced and published by governmental agencies, academic institutions, and other groups, which are not available in the academic press). Inclusion criteria were as follows: (i) adults and children or communities living within the Mediterranean coastal countries; (ii) interventions to promote the use of marine environments or the consumption of seafood (this includes exposure to marine pollutants and toxins); health and wellbeing impacts (both positive and negative) of recreational activities at sea and arising from seafood consumption; health and wellbeing impacts of living by and on marine environments; benefits of marine organisms with bioactive potential to human health (discovery of new drugs). The exclusion criteria included: (i) studies that did not specifically document or measure the health or wellbeing outcomes that people receive from exposure to, or interventions with, the marine environment; (ii) studies that focused solely on the health benefits or costs from fresh water; (iii) theoretical studies or models; (iv) commentaries and editorials.

While more than 100 key documents were reviewed, the literature review should not be considered to be a formal systematic activity, given that only key documents providing examples of the issues identified were selected. In addition, websites of topic-related projects (e.g., SOPHIE, BlueHealth, Blue Gym, ECsafeSEAFOOD, SEAFOOD^TOMORROW^, and NSF/NIEHS/NOAA’s OHH Initiative) and organizations dealing with OHH issues (e.g., the University of Exeter Medical School in the UK, the Center for Climate, Health, and the Global Environment at the Harvard T.H. Chan School of Public Health in the US, the Barcelona Institute for Global Health (ISGLOBAL) in Catalonia (Spain), and the Centre of Expertise for Waters in Scotland) were scrutinized.

## 5. Results

### 5.1. Geographical Dimensions

One of the agreements reached among the stakeholders was that, while the Chair should be locally and regionally rooted, it should also have an international dimension and scope. Thus, several international and regional/local level cornerstone strategies were endorsed by the Chair (Supplementary Table S2). At the international level, these included key integrated marine and health policies implemented in Europe and worldwide that aim to provide a coherent approach to human health and environmental issues, including marine ecosystems, and that increase coordination among different policy and research areas [46]. Within the local/regional context, the Chair endorsed several policies that have been implemented in Catalonia with the aim of managing environmental and human health in a more interconnected way (Supplementary Table S2). However, these policies are currently biased towards the urban environment and terrestrial and freshwater environments, with very little emphasis placed on marine and coastal environments.

In this context, the Chair supported the introduction of global maritime policies on the local/regional scale, coping with the bias towards terrestrial environments, basically by contributing to the 2030 Maritime Strategy of Catalonia, which establishes a maritime policy that considers all activities that have an impact on Catalan maritime space. The Chair contributed, among other issues, to the structure of a scientific network in OHH in Catalonia; to the development of responsible maritime tourism and fishing activities and co-management plans; and to the dissemination of the health benefits of the Mediterranean diet and the practice of activities in blue spaces. These contributions were recognized with an award given by the Autonomous Government of Catalonia to the Chair in 2019.

### 5.2. Current and Emerging OHH Issues

**Figure 4:**
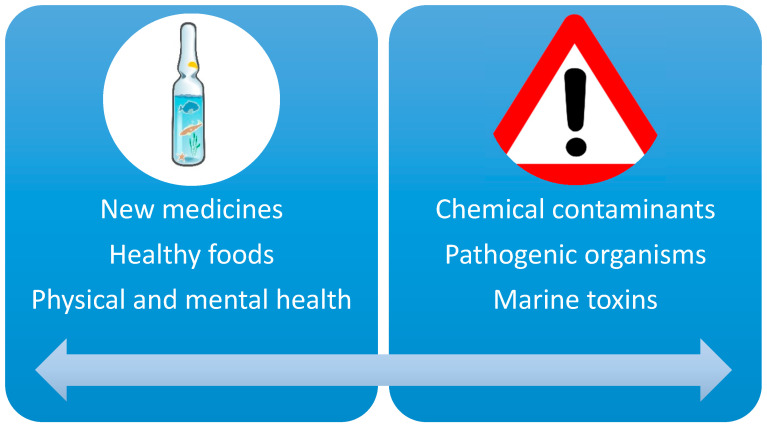
The current and emerging OHH issues identified by the Chair during the implementation phase: health benefits (**left**) and health risks (**right**).

The current and emerging OHH issues identified by stakeholders were initially separated by academic experts into three main topics (Fish and Health; Leisure, Health, and Wellbeing; and Medicines from the Sea) and two groups (the benefits provided by marine ecosystems for human health and wellbeing and the health risks from these marine ecosystems). Among the benefits (Figure 4, left), the main issues identified were: (i) the bioactive potential of marine organisms as a source of new medicines; (ii) healthy foods from the sea (“the healthy Mediterranean diet”); and (iii) the physical and mental health benefits linked to living near the coast and/or taking part in leisure activities at sea. Among the main health risks to humans (Figure 4, right) are those that come via exposure to seawater or from consuming seafood products, and are grouped as follows: (i) chemical contaminants; (ii) pathogenic organisms; and (iii) marine toxins.

The negative health and environmental effects of certain locally expanding activities, such as leisure boating and cruising, linked to the population increase and mass tourism in the Mediterranean since the 1950s were also considered. Interestingly, during this same time period, the SOPHIE Project created a Strategic Research Agenda [14] via an expert and consultative process which identified three key topics to cement OHH as a field of study in Europe: sustainable seafood and healthy people; blue spaces, tourism, and wellbeing; and marine biodiversity, medicine, and biotechnology.

The most important issues among these benefits and risks for the context of developed Mediterranean countries were prioritized and sorted into two lists, the first consisting of marine environmental issues (or challenges) with associated well-defined public health challenges (Supplementary Table S3), and the second of major human health issues (or challenges) not directly linked to any particular environmental challenge but in which ocean and coastal preservation can play an important role (Supplementary Table S4). Specific challenges facing Northern African countries were not considered because developing countries have different socioeconomic characteristics and health pathways [16].

### 5.3. The Positive Outcomes and Lessons Derived from the Chair

The Chair demonstrates the capabilities of local, bottom-up, transdisciplinary, and trans-sector models for engaging stakeholders in the research and management of emerging OHH issues, which can be implemented, not only locally in other coastal regions of the Mediterranean, but also in the local contexts of other seas/oceans.

The Chair is a good example of how such an approach can help marine science and medical disciplines work together towards a final goal under the conviction that, the earlier the most effective preventive measures are implemented locally and regionally, the more health benefits and fewer risks linked to the sea will be enjoyed by citizens (and vice versa). Our sense from the discussions held with the Advisory Board and the Follow-Up Committee during the annual meetings in 2018 and 2019 is that stakeholder involvement increased the commitment of local citizens towards marine conservation as a tool to preserve human health and wellbeing (however, this is a anecdotal evidence; a full qualitative evaluation of the nature of this commitment has still to be made). Furthermore, stakeholder involvement in identifying and prioritizing challenges has facilitated their subsequent shared commitment to marine environmental and social justice and to ethical research practices, which is essential to promote a sustainable and healthy partnership approach in health and environmental research [47,48].

Another positive outcome is that the consideration of human health impacts has added a human dimension to local people’s understanding of ongoing local environmental impacts. While the first was easily accepted by all stakeholders, some environmental impacts, such as the impact of fishing, have been a source of controversy amongst them.

Last, despite the fact that transdisciplinarity is still considered to be an emergent, loosely defined approach that lacks traction in practice [49,50], the Chair seems to have been able to successfully incorporate transdisciplinary studies that explore the main trade-offs between the benefits and risks associated with the seas. In the Mediterranean context, these trade-offs can be summarized as:

(i) the promotion of the marine environment for recreational use to treat or possibly decrease the prevalence of mental disorders, non-communicable diseases (NCDs) that are the leading cause of disability in many Western European countries [18], vs. the need to prevent overcrowding and the consequent environmental degradation of blue spaces, including the impact of ecotourism activities such as scuba diving in MPAs [37];
(ii) the promotion of seafood consumption as a source of high-quality nutrients in the Mediterranean diet vs. the decline of fish stocks due to overfishing and climate change in the Mediterranean, the health risks posed by contaminated seafood, and changing dietary preferences [36];
(iii) the exploration of marine species with bioactive potential to discover new drugs vs. the conservation of these species and overall biodiversity. Although only small quantities of biological resources are harvested, exploring the ocean for new biotechnological products can cause significant environmental disturbance with potential adverse effects [14]. Furthermore, the growing importance and value attached to alternative medicine based on animal and plant products in some countries may increase the risk of the extinction of vulnerable species.

**Figure 5:**
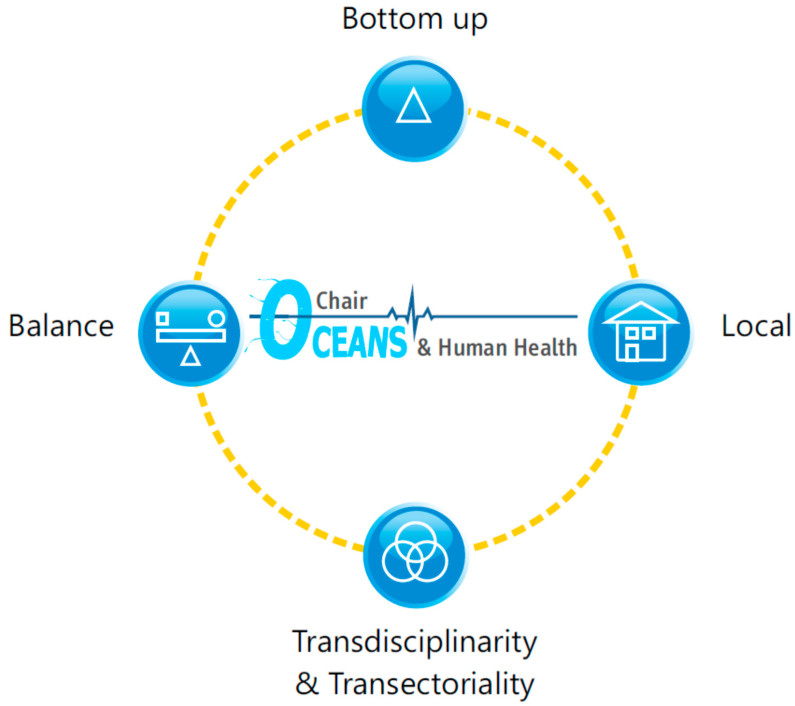
The relevant principles resulting from the OHH Chair to foster new participatory process in oceans and human health.

Drawing on the example provided by the Chair, four principles were established by the academic and non-academic experts through observations to ensure better participatory processes in the OHH field, which are summarized in Table 1 and schematized in Figure 5.

### 5.4. Drawbacks and Difficulties Encountered

Despite the positive outcomes achieved using the approach adopted by the Chair, several difficulties and drawbacks have been identified. One of the major difficulties was the limited support for implementing measures that can have a long-term impact on the sustainable development of our coasts and seas and benefit public health, but do not have a clear impact in the short term, such as shifting from mass tourism to ecotourism. In particular, not all local businesses have accepted these measures given the potential economic risks of such changes.

Another obstacle regarding the trade-offs was the gap between scientific and citizen practices. Not all citizens understood the requirements of scientific research, and some non-experts have raised the issue of alternative methods and therapies related to the sea and its components which cannot be addressed effectively or accepted from a scientific point of view because they do not currently have supporting data, validation, or sufficient scientific scrutiny, or because no medical guidelines have been established for them. For example, halotherapy, an alternative but controversial treatment involving breathing salty air, which has been proposed to treat respiratory conditions and allergies [51]; the use of sunlight therapy (heliotherapy) to treat certain medical conditions such as psoriasis and atopic dermatitis [52]; and the potential health benefits of drinking sea water [53]. Given that some of these alternative therapies can have serious side effects [54,55], they have not been accepted by the Chair’s experts (i.e., they were excluded from the medical praxis), creating frustration among a minority of citizen stakeholders. These examples show that participatory processes also have limits that must be carefully considered.

Another challenge that was encountered, which was anticipated in the first years, was the difficulty in attracting large and diverse (all ages and socio-economic conditions) publics, meaning that the Chair’s influence has only reached a relatively small portion of the local population. Most of the citizens (80% of the total) that have engaged in the activities up to now have a relatively good educational level (high school or higher) and have direct (professional) or indirect (as a hobby) links with marine and health issues. Therefore, there is a need to target other stakeholder communities to incorporate their different experiences and understandings through working with local businesses and sport clubs, the town’s social services, and the children in local schools.

Another drawback is the controversy surrounding some environmental issues, such as whether fishing activities can become sustainable and whether some ecotourism activities, such as scuba diving, can be considered as “ecofriendly” in MPAs.

## 6. Discussion

The Chair is an example of how institutional support for standing public involvement groups can provide conduits for connecting the public with policy makers and academic institutions [2]. The Chair offers an example of how a local, bottom-up approach can encourage widespread, high-quality local responses to big challenges linking marine ecosystem health with the health and wellbeing of citizens.

The Chair approach provides a model that shows that the simultaneous improvement of marine environmental and public health needs to be rooted in local circumstances, where community knowledge, engagement, and commitment are key. Local stakeholders have sound knowledge of the environmental and health challenges of an area, which can be integrated (with some exceptions, such as those related to alternative medicines) into and embraced by research approaches that aim to address large-scale problems. Furthermore, the Chair has created an environment for creativity and innovation, with the flexibility and space required to rethink and develop novel approaches within the OHH field of study.

It must be considered that contradictions between the bottom-up and top-down directions of management in representative democracies remain [8], and that all kinds of participatory processes can fail [56,57]. Nevertheless, many examples have demonstrated that the risk of failure is lower if local contextual issues are considered [25,55]. The Chair approach contributes to the idea that, instead of assuming that best practice solutions can be exclusively prescribed centrally or nationally, local communities should be supported in developing and delivering solutions that reflect local needs and engage citizens, with the aim of tackling national and global concerns more effectively [25]. Given that OHH is part of a wider environment and health research field within the “Planetary Health” and “One Health” approaches [58,59], local initiatives like the Chair must also be embedded within a global perspective.

The urgent and increasingly costly nature of many of the health and marine environmental challenges that were identified, such as the ongoing degradation of marine habitats and resources and increasing mental and physical health problems, require solutions that are more effective in engaging and involving diverse publics. Considering the rising environmental and healthcare costs in developed countries [16], the replication of the Chair in other regions may support public finance savings if the interactions between marine ecosystems and human activities (e.g., the preservation of fish stocks that constitute a source of healthy food needed to help prevent some illnesses) are well managed. The initiatives promoted by the Chair, which focus on health promotion and disease prevention rather than treatment, are important in a general context where the infrastructure of many national health systems is still geared towards treating illnesses and the preventative health agenda remains comparatively under-supported, despite the evidence suggesting that the latter could significantly drive down costs [16].

The Chair’s initiatives could contribute to a preventive health agenda through addressing the major behavioral risk factors common to many NCDs, such as cardiovascular disease and cancer (the two most important NCDs in terms of deaths), including improving diets, increasing physical activity, and reducing obesity [16]. In this sense, the three main topics identified by local stakeholders (Fish and Health; Leisure, Health, and Wellbeing; and Medicines from the Sea) constitute the basis for the development of further studies that consider both the benefits and risks for human health and wellbeing. Furthermore, the stakeholders’ involvement can help tackle the rising inequalities in access to some expensive seafood species and recreational activities, such as sailing and scuba-diving, through the integrated and sustainable management of marine ecosystems and the promotion of fair and equitable sharing and access to sea resources and spaces.

The challenges facing the Chair are not unique to the region (Catalonia), country (Spain), or the Mediterranean area, but they are transboundary. Therefore, the lessons learned from the Chair in the local context may be further replicated in other regions. Nevertheless, it must be considered that, in many cases, these processes are powerful in very specific contexts [25], and therefore they can only be replicated in some specific places once the local context has been well integrated. Hence, the Chair is not a transferable model that fits all research, institutions, communities, regions, and countries, but is a flexible approach to fostering and maintaining public involvement, engagement, and participation within the OHH field of study.

## 7. Conclusions

Overall, we judge the creation of the Chair, particularly the ongoing involvement of stakeholders in the Chair, to have been successful and impactful, helping to shape and prioritize the research and policy agendas in the OHH field. The Chair has become a local hub that stimulates innovative and proactive thinking in OHH topics, while developing co-production and genuine knowledge exchange lessons for supporting heathy marine environments and people. The Chair has clearly led the engagement of stakeholders in defining research priorities related to OHH, which ensures smooth local management decisions and policy implementation. Furthermore, the Chair has successfully integrated most of its experts in OHH from the most prestigious institutions in Catalonia. Overall, the following specific conclusions can be drawn:

- Bottom-up approaches can engage communities in oceans and human health challenges;
- Responses to these challenges need a trans-sectorial and transdisciplinary vision;
- Improvement in these challenges should be rooted in local circumstances;
- The Oceans and Human Health Chair shows how diverse stakeholders can co-create together towards their health and the health of their local environment.

The Chair can contribute decisively, not only to gathering new scientific knowledge on OHH topics, but also to the design of research questions and the sharing of new knowledge among stakeholders, while helping the administrations set up sound policies regarding the protection of the health of both the marine environment and citizens.

## Figures and Tables

**Table 1:** The basic principles established by the Chair to ensure better participatory processes in the OHH field, together with a short description.

Principle	Description
Bottom-up approach	Instead of assuming that the best solutions need to be determined, prescribed, driven, or “authorised” in some way from the top, policy makers should create more opportunities for communities to develop and deliver their own solutions.
Think local	The need to think local to create the conditions for change to happen on a global or national scale. Governments should look for solutions beyond established large international or national organizations and consider local organizations, including primary care centers, NGOs, patients’ organizations, and citizens themselves, working hand in hand with experts.
Transdisciplinary and trans-sectorial approach	Cross-cutting actions among experts from several institutions (universities, research centers, local and regional governments, NGOs) and the community (patients’ organizations, fishers, the tourism industry, etc.) can be effective and efficient tools to detect needs and can be adopted as a way to study complex problems in the field of OHH.
Balance the many voices	The need to balance these voices among different stakeholders. The Chair approach stresses the importance of considering citizen preferences and perceptions, facilitating dialogue between citizens and experts, and reaching consensus regarding controversial environmental and health issues to bring about positive health and environmental change. This balance takes trust and dedicated time.

## Supplementary Materials

The following are available online at https://www.mdpi.com/1660-4601/17/14/5078/s1, Tables S1–S4. Table S1: List of public engagement and participation activities carried out by the Chair, 2018–2020. Table S2: International and local/regional cornerstone strategies endorsed by the Chair, together with a short description of each. Table S3: List of major environmental issues together with their factors and drivers, and associated public health challenges identified by the stakeholders during the implementation phase of the Chair. Key references used are shown. Table S4: List of major human health issues and associated goals within the framework of OHH proposed by the Chair’s stakeholders. Key references used are shown.
