# Supplementary material for: The Roses Ocean and Human Health Chair: A New Way to Engage the Public in Oceans and Human Health Challenges
Source: Int J Environ Res Public Health. 2020 Jul 14;17(14):5078. doi: 10.3390/ijerph17145078 (PMC7400534; doi:10.3390/ijerph17145078)
Supplement: Supplementary file 1 [file ijerph-17-05078-s001.pdf]

SUPLEMENTARY MATERIAL

Table S1. List of public engagement and participation activities carried out by the Chair, 2018-2020.

Public engagement	Description
Workshops	Two major workshops held in 2010 and 2018, coordinated by the University of Girona
Science open days	Conferences and demonstrative activities held in 2018 and 2019 (including demonstrative laboratory sessions and demonstrative activities at sea)<br>Co-organized with the Catalan Institute of Oncology and the Catalan Health Institute
Science festivals	Lectures as part of events organized by other institutions in different localities in 2018 and 2019
TV and radio	Participation in local and regional programmes (interviews)
Newspapers	Publication of articles in several local and regional newspapers
Other publications	Publication of other dissemination materials such as guides (e.g., Omega 3 Guide and the Maritime Recreational Activities and Human Health guide); labels and banners related to preventing human health issues in connection with the conservation of the marine ecosystems; certificates to acknowledge stakeholders' engagement in the Chair's activities
Website	Creation of the Chair's website (www.oceanshealthudg.edu) containing information about the Chair's structure and goals and a summary of the OHH topics covered
Social media	Creation of the Chair's social media channels (Twitter, Facebook, Instagram, and YouTube) to quickly disseminate news to the public
Exhibitions	Creation of a touring, temporary exhibition illustrating how marine ecosystems are linked to human health and wellbeing.<br>Collection of explanatory panels, photos, animals, and chemical compounds<br>The mobile OHH, which can be moved from town to town, to illustrate how life evolved from water
Films	Elaboration of a full documentary film about OHH (to be presented in 2020) and short videos about several OHH topics
Conferences	Conferences for particular groups (high school students, the elderly, environmental and health professionals, etc.) within the framework of specific conference series on health and marine environmental issues organized by other institutions at the regional, national, and international levels
Other	Preparation of promotion material for the public (stickers, T-shirts, etc.)

Public participation	Description
	Research study in Cap de Creus to assess whether snorkelling and scuba diving at sea may contribute to the improved wellbeing of healthy people and oncology patients<br>Public perception questionnaires (open and closed ended questions) and focus groups

Research studies on the effects of maritime recreational activities on human health and well-being	In collaboration with the Catalan Health Institute, the Catalan Oncology Institute, the Roses Against Cancer Foundation, and local scuba diving clubs<br>Goal: development of "Blue Prescriptions" by local community health practitioners, considering blue spaces as a "health asset" (i.e., a resource that enhances the ability of individuals, communities, and populations to maintain and sustain health and wellbeing). "Blue Prescriptions" are health professionals' advice to patients to be physically active in or near the sea, considering the salutogenic effects of blue spaces (sea and coast), which are similar to the "green prescriptions" implemented in some countries such as New Zealand, Scotland, and Japan.
	Research study in Cap de Creus on the complex links between several marine recreational activities and human health and wellbeing. The conclusions drawn were based on published studies and the personal experience of qualified, professional instructors in the area of Cap de Creus in response to open-ended questionnaires.<br>In collaboration with the MedPAN Network of MPAs in the Mediterranean and local companies and associations offering leisure activities at sea (e.g., kayaking, swimming, sailing, scuba diving, etc.).<br>Goal: to increase awareness among stakeholders of the existing relationships between the marine recreational activities taking place in the MPA, and human health and citizen wellbeing.
	Doctoral Thesis and Final Bachelor's Degree Projects on the effects of maritime and coastal activities such as kayaking and coastal walking on citizen health and wellbeing.<br>Final Bachelor's Degree Project on Tetrodoxins (TTXs) in pufferfish.<br>Co-financed by Tossa de Mar town council and the Chair.<br>In collaboration with local stakeholders (scuba diving clubs, kayaking associations, fisher's associations, etc.) and IRTA.<br>Goal 1: to develop a new tourist model more based on sustainable and healthy maritime and coastal leisure activities, while helping to improve the tourism offer during the low season<br>Goal 2: to contribute to the knowledge of TTXs in pufferfish
Research study on the self-monitoring of health variables	Use of smart watches (wearable tech) to enable ocean and coastal users to self-monitor health variables, and to support bi-directional communication with health care providers<br>In collaboration with the Catalan Health Institute, the Catalan Oncology Institute, and local snorkelling/scuba diving clubs<br>Goal: to assess whether snorkelling at sea may contribute to improved health variables such as heart frequency, blood pressure, and hours of sleep in oncology patients
Biological studies with health repercussions	Research study to analyse the omega-3 fatty acids in the most valued fishery species caught in the Costa Brava region (Northern Catalonia). Search for alternative or complementary sources of omega 3 fatty acids. Review of the benefits of omega-3 for human health (e.g., cardiovascular risk and cancer). Evaluation of omega 3 fatty acids in fish prey as an indicator of habitat quality. Communication of the results of the project to the public and the stakeholders involved via the "Omega-3 Guide to the Costa Brava" and workshops

	In collaboration with the Institute of Marine Sciences (ICM-CSIC), the D.G Fisheries of the Government of Catalonia, the Fisheries Local Action Group (FLAG) Costa Brava, the Catalan Institute of Oncology, local professional fisheries. associations, fishmongers, fish restaurants, and fish processing plants<br>Goal: to evaluate the health risks in seafood (parasites and heavy metals)
Social studies with health repercussions	Research study on the social determinants of health, including equitable access to marine resources among people from different ethnic groups<br>In collaboration with the Social Anthropology Dept. of the Autonomous Barcelona University, the Fisheries Local Action Group (FLAG) Costa Brava, local professional fisheries associations, and fishmongers<br>Goal: to integrate the sociocultural issues in the OHH field

Table S2. International and local/regional cornerstone strategies endorsed by the Chair, together with a short description of each.

Level	Cornerstone strategy	Description	References
International	One Health and Planetary Health approaches	Two worldwide strategies for expanding interdisciplinary collaborations and communications in all aspects of health care for humans, animals, and the environment to safeguard the health of the environment and humans.	Lerner et al., 2017<br>Hill-Cawthorne, 2019
	Precautionary principle	A worldwide strategy initially designed for approaching issues of potential environmental risks when extensive scientific knowledge on the matter is lacking, but can also be extended into public health	Kriebel et al., 2001<br>Sand, 2000
	Sustainable Development Goals of the UN's 2030 Agenda for Sustainable Development.	A worldwide strategy, the goals of which include the conservation of the seas and oceans, the sustainable use of the marine resources, the promotion of a healthy life, the preservation of people's wellbeing, and the reduction of inequalities. In particular SGD no. 14: Conservation and sustainable use of the oceans, seas, and marine resources.	The Sustainable Development Agenda – United Nations Sustainable Development, 2020
	EU's marine and health policies	Health 2020 European Policy Framework and Strategy for the 21st Century<br>Marine Strategy Framework Directive<br>Bathing Water Directive<br>Maritime Spatial Planning Directive<br>Blue Growth Strategy<br>Food 2030<br>Strategic Research Agenda (SRA) for Oceans and Human Health in Europe	H2020 SOPHIE Consortium, 2020<br>Blue growth ǀ Maritime Affairs, 2020<br>Maritime spatial planning ǀ Maritime Affairs, 2020<br>Bathing water Directives — European Environment Agency, 2020<br>Law - EU Coastal and Marine Policy - Environment - European Commission, 2020<br>WHO Regional Office for Europe, 2013
Regional/local	Marine and health policies in Catalonia	2030 Maritime Strategy of Catalonia<br>Catalan Strategy on the marine environment and biodiversity 2018-2030<br>Health Promotion Plan (PAAS)<br>Catalan Health Plan 2016-2020<br>Local Public Health Plan (PINSAP)- COMSalut Project<br>Food Security Plan 2017-2021<br>Co-management model of the professional fisheries in Catalonia.	2030 Maritime Strategy of Catalonia, 2020<br>Pla de seguretat alimentària de Catalunya 2017-2021. Agència Catalana de Seguretat Alimentària, 2017

	Pla Interdepartamental i Intersectorial de Salut Pública (PINSAP). Agència de Salut Pública de Catalunya, 2018<br>Pla de salut 2016-2020. Departament de Salut, 2016<br>PAAS. Agència de Salut Pública de Catalunya (ASPCAT), 2020<br>DECRET 118/2018 sobre el model de governança de la pesca professional a Catalunya (DOGC 7647 21 June 2018).

References:

2030 Maritime Strategy of Catalonia. 2020. http://agricultura.gencat.cat/web/.content/08-pesca/politica-maritima/enllacos-documents/fitxers-binaris/ESTRATEGIA-MARITIMA-2030-Pla-2018-2021_EN.pdf.

Bathing water Directives — European Environment Agency. 2020. Retrieved May 25, 2020, from https://www.eea.europa.eu/themes/water/europes-seas-and-coasts/assessments/state-of-bathing-water/bathing-water-directives

Blue growth | Maritime Affairs. 2020. Retrieved May 25, 2020, from https://ec.europa.eu/maritimeaffairs/policy/blue_growth_en

H2020 SOPHIE Consortium 2020. A Strategic Research Agenda for Oceans and Human Health in Europe. H2020 SOPHIE Project. Ostend, Belgium. ISBN: 9789492043894 DOI: 10.5281/zenodo.3696561 Retrieved May 25, 2020, from https://ec.europa.eu/info/news/strategic-research-agenda-oceans-and-human-health-europe-2020-mar-31_en

Hill-Cawthorne, G. A., 2019. One Health/EcoHealth/Planetary Health and their evolution. In One Planet, One Health (pp. 1–20). Sydney University Press. http://doi.org/10.2307/j.ctvggx2kn.6

Kriebel, D., Tickner, J., Epstein, P., Lemons, J., Levins, R., Loechler, E. L., Quinn, M., Rudel, R., Schettler, T., Stoto, M., 2001. The Precautionary Principle in Environmental Science (Vol. 109). Retrieved from http://ehpnet1.niehs.nih.gov/docs/2001/109p871-876kriebel/abstract.html

Law - EU Coastal and Marine Policy - Environment - European Commission. (2020). Retrieved May 25, 2020, from https://ec.europa.eu/environment/marine/eu-coast-and-marine-policy/marine-strategy-framework-directive/index_en.htm

Lerner, H., and Berg, C., 2017. A Comparison of Three Holistic Approaches to Health: One Health, EcoHealth, and Planetary Health. *Front. Vet. Sci.*, *4*, 163. http://doi.org/10.3389/fvets.2017.00163

Maritime spatial planning | Maritime Affairs. (2020). Retrieved May 25, 2020, from https://ec.europa.eu/maritimeaffairs/policy/maritime_spatial_planning_en

PAAS. Agència de Salut Pública de Catalunya (ASPCAT). 2020. Retrieved May 25, 2020, from http://salutpublica.gencat.cat/ca/sobre_lagencia/Plans-estrategics/PAAS/

Pla de salut 2016-2020. Departament de Salut. 2016. Retrieved May 25, 2020, from https://salutweb.gencat.cat/ca/el_departament/Pla_salut/pla-de-salut-2016-2020/
Pla de seguretat alimentària de Catalunya 2017-2021. Agència Catalana de Seguretat Alimentnària, 2017. Retrieved May 25, 2020, from https://scientiasalut.gencat.cat/handle/11351/3225?locale-attribute=en
Pla Interdepartamental i Intersectorial de Salut Pública (PINSAP). Agència de Salut Pública de Catalunya (ASPCAT). 2018. Retrieved May 25, 2020, from http://salutpublica.gencat.cat/ca/sobre_lagencia/Plans-estrategics/pinsap/
Sand, P. H., 2000. The precautionary principle: A European perspective. *Hum. Ecol. Risk Assess.*, *6*(3), 445–458. http://doi.org/10.1080/10807030091124563
The Sustainable Development Agenda – United Nations Sustainable Development. 2020. Retrieved May 25, 2020, from https://www.un.org/sustainabledevelopment/development-agenda/
WHO Regional Office for Europe. 2013. Health 2020: A European policy framework and strategy for the 21st century. Copenhagen: World Health Organization, Regional Office for Europe, UN City, Marmorvej 51, DK-2100 Copenhgen, Denmark. Retrieved May 25, 2020, from http://www.euro.who.int/__data/assets/pdf_file/0011/199532/Health2020-Long.pdf

Table S3. List of major environmental issues together with their factors and drivers, and associated public health challenges identified by the stakeholders during the implementation phase of the Chair. Key references used are shown.

Environmental issue	Environmental factors/drivers	References	Public health challenge	References
Decline of local fishery resources	Omega 3 fatty acids and other micronutrients	Gow et al., 2014<br>García-Moreno et al., 2013	Loss of local supply of omega 3 fatty acids, which provide various health benefits for consumers.<br>Search for alternative or complementary sources of omega 3 fatty acids.<br>Consideration of health benefits of seafood beyond mere consumption and protein.<br>Preservation and better use of wild stocks of omega 3 fatty acids to optimize their utilization for human consumption.<br>Better understanding of the linkages between fisheries, nutrition, and health.	Hicks et al., 2019;<br>Leppo et al., 2013;<br>Lloret et al., 2016<br>Román et al., 2019
Decrease in condition/quality of seafood species	Heavy metals<br><br>Persistent Organic Contaminants	Kim et al., 2009<br>Trainer et al., 2015<br>Dallinger et al., 1987	Current and emerging health risks of seafood consumption leading to acute or chronic health diseases such as nervous system diseases, allergies, gastrointestinal diseases, reproductive system	Berdalet et al., 2016;<br>González et al., 2018;

	Microplastics<br><br>Toxins in seafood (e.g. shellfish) from Harmful Algal Bloom (HABs) species (certain dinoflagellates and cyanobacteria).<br><br>Known (saxitoxins, microcystins, anatoxins, etc.) and emerging toxins (Tetrodotoxins and ciguatoxins)<br><br>Pathogenic organisms (vectors): Bacteria, virus and parasites	Smith et al., 2018<br>Johnson et al., 2013	diseases, immune diseases, and chronic inflammation (which increases the risk of cancer).	González et al., 2019;<br>Prata et al., 2020;<br>Trabalón et al., 2017;<br>Vilavert et al., 2017
Air, water and noise pollution from maritime recreational activities	Maritime cruising<br>Leisure motor boating	Perić et al., 2016<br>Maragkogianni et al., 2015<br>Rako et al., 2013	Air and water pollution from leisure boats and cruise ships (e.g., bad air quality on the decks of cruise ships and in the vicinity of ports and berths) may affect both users' health and that of other citizens (e.g., swimmers, local residents, etc)<br><br>Higher risk of infections on board cruise ships	Egardt et al., 2018;<br>EU Commission, 2007;<br>Murray et al., 2011
Effects of tourism overcrowding	Diving<br>Leisure boating<br>Sunbathing on the beach	Venturini et al., 2016<br>Garrod et al., 2008<br>Jędrzejczak, 2004<br>Bujosa et al., 2015	Sunbathing, which may be linked to excessive sun exposure (increased cancer risk), and non-exercising (increase in overweight/obese people)	Camp et al., 2012;<br>Lucrezi et al., 2017;<br>Venturini et al., 2018

			Sea overcrowding by users can decrease satisfaction with the experience and hence the potential wellbeing effect of the activity	
Degradation of the quality of the coastal and marine environment	Domestic, industrial and agriculture waste, including organic and inorganic chemicals and plastics.<br>HABs.<br>Pathogens (resistant bacteria, virus, etc.)	EEA, 1999<br>Valavanidis, 2018	Quality reduction or loss of blue spaces where citizens can practice healthy outdoor activities. Specific health risks for certain sea users (e.g., swimmers and surfers) posed by exposure to toxic algae (e.g., *Ostreopsis*) and antibiotic resistant strains of bacteria (e.g., E. coli).	Borja et al., 2020;<br>Vila et al., 2016
Impacts of global change on vulnerable marine species with bioactive potential	Loss of biodiversity / Risk of extinction<br>Mass mortalities of certain taxa<br>Oxygen limitation and acidification<br>Declining populations	Casoli et al., 2017;<br>Peters, 2019;<br>Verdura et al., 2019	Loss of the opportunities that the bioactive potential of these species represent for discovering new medicines (e.g., antiviral, antifungal, antibacterial, and particularly antitumoral) within the framework of marine biotechnology.	Carreño and Lloret, 2019
Marine protected areas (MPAs) as a management tool for preserving the marine ecosystems and promoting better health and wellbeing.	Protection of fish stocks.<br>Protection of blue spaces.<br>Protection of species with bioactive potential.<br>Protection of biodiversity.	EEA, 2015<br>MARINE PROTECTED AREAS – MedPAN, 2020	Endure the availability of local stocks of omega 3.<br>Ensure the preservation of blue spaces.<br>Ensure the preservation of species with possible pharmacological interest.<br>Ensure the protection of marine species used in fundamental biological and medical research.	Francour et al., 2001;<br>Martin et al., 2017;<br>Rees et al., 2015;<br>Wells et al., 2016

References:

Berdalet, E., Fleming, L. E., Gowen, R., Davidson, K., Hess, P., Backer, L. C., Moore, S. K., Enevoldsen, H., Hoagland, P., 2016. Marine Harmful Algal Blooms, Human Health and Wellbeing: challenges and opportunities in the 21st century. J. Mar. Biolog. Assoc. U.K., 96 (Special Issue 01): 61-96, doi:10.1017/S0025315415001733

Borja, A., White, M. P., Berdalet, E., Bock, N., Eatock, C., Kristensen, P., Leonard, A., Lloret, J., Pahl, S., Parga, M., Prieto, J. V., Wuijts, S., Fleming, L. E., 2020. Moving Toward an Agenda on Ocean Health and Human Health in Europe. Front. Mar. Sci., 7, 37. http://doi.org/10.3389/fmars.2020.00037

Bujosa, A., Riera, A., and Pons, P. J., 2015. Sun-and-beach tourism and the importance of intra-destination movements in mature destinations. Tour. Geogr., 17(5), 780–794. http://doi.org/10.1080/14616688.2015.1093538

Camp, E., and Fraser, D., 2012. Influence of conservation education dive briefings as a management tool on the timing and nature of recreational SCUBA diving impacts on coral reefs. Ocean Coast. Manag., 61, 30–37. http://doi.org/10.1016/J.OCECOAMAN.2012.02.002

Casoli, E., Nicoletti, L., Mastrantonio, G., Jona-Lasinio, G., Belluscio, A., Ardizzone, G. D., 2017. Scuba diving damage on coralligenous builders: Bryozoan species as an indicator of stress. Ecol. Indic., 74, 441–450. http://doi.org/10.1016/J.ECOLIND.2016.12.005

Dallinger, R., Prosi, F., Segner, H., Back, H., 1987. Contaminated food and uptake of heavy metals by fish: a review and a proposal for further research. Oecologia, 73(1), 91–98. http://doi.org/10.1007/BF00376982

EEA. 1999. *State and pressures of the marine and coastal Mediterranean environment*. *European Environment Agency*. http://doi.org/http://europa.eu.int

EEA. 2015. *Marine protected areas in Europe's seas. An overview and perspectives for the future.* http://doi.org/10.2800/99473

Egardt, J., Mørk Larsen, M., Lassen, P., Dahllöf, I., 2018. Release of PAHs and heavy metals in coastal environments linked to leisure boats. Mar. Pollut. Bull., 127, 664–671. http://doi.org/10.1016/J.MARPOLBUL.2017.12.060

EU Commission. 2007. Environmental Impacts of Recreational Boating. Retrieved from http://ec.europa.eu/environment/integration/research/newsalert/pdf/87na2_en.pdf

Francour, P., Harmelin, J.-G., Pollard, D., Sartoretto, S., 2001. A review of marine protected areas in the northwestern Mediterranean region: siting, usage, zonation and management. Aquat. Conserv., 11(3), 155–188. http://doi.org/10.1002/aqc.442

García-Moreno, P. J., Pérez-Gálvez, R., Morales-Medina, R., Guadix, A., Guadix, E. M., 2013. Discarded species in the west Mediterranean sea as sources of omega-3 PUFA. Eur. J. Lipid Sci. Technol., 115(9), 982–989. http://doi.org/10.1002/ejlt.201300021

Garrod, B., and Gössling, S., 2008. New Frontiers in Marine Tourism: Diving Experiences, Sustainability, Management. *New Frontiers in Marine Tourism,* 3–28. http://doi.org/10.1016/b978-0-08-045357-6.50004-8

González, N., Calderón, J., Rúbies, A., Timoner, I., Castell, V., Domingo, J.L., Nadal, M., 2019. Dietary intake of arsenic, cadmium, mercury and lead by the population of Catalonia, Spain: Analysis of the temporal trend, Food Chem. Toxicol., 132, 110721.

González, N., Marquès, M., Nadal, M., Domingo, J.L., 2018. Levels of PCDD/Fs in foodstuffs in Tarragona County (Catalonia, Spain): Spectacular decrease in the dietary intake of PCDD/Fs in the last 20 years. Food Chem. Toxicol., 121, Pages 109-114.

Gow, R. V., and Hibbeln, J. R., 2014. Omega-3 fatty acid and nutrient deficits in adverse neurodevelopment and childhood behaviors. Child Adolesc. Psychiatr. Clin. N. Am. W.B. Saunders. http://doi.org/10.1016/j.chc.2014.02.002

Hicks, C., Cohen, P., Graham, N., Nash, K., Allison, E., D'Lima, C., Mills, D. J., Roscher, M., Thilstead, S. H., Thorne-Lynam, A. M. M., 2019. Harnessing global fisheries to tackle micronutrient deficiencies. Nature, 574, 95–98.

Jędrzejczak, M., 2004. The modern tourist's perception of the beach: Is the sandy beach a place of conflict between tourism and biodiversity? Coastline Reports, 2(2004), 109–119. Retrieved from http://balticeucc.databases.eucc-d.de/files/documents/00000380_Jedrzejczak.pdf

Johnson, L. L., Anulacion, B. F., Arkoosh, M. R., Burrows, D. G., da Silva, D. A. M., Dietrich, J. P., Myers, M. S., Spromberg, J., Ylitalo, G. M., 2013. Effects of Legacy Persistent Organic Pollutants (POPs) in Fish-Current and Future Challenges. *Fish Physiol.*, *33*, 53–140. http://doi.org/10.1016/B978-0-12-398254-4.00002-9

Kim, J. H., Lee, K. J., Suzuki, T., Kim, C. M., Lee, J. Y., Mok, J. S., Lee, T. S., 2009. Identification of tetramine, a toxin in whelks, as the cause of a poisoning incident in Korea and the distribution of tetramine in fresh and boiled whelk (Neptunea intersculpta). J. Food Prot., 72(9), 1935–40. Retrieved from http://www.ncbi.nlm.nih.gov/pubmed/19777897

Leppo, K., Ollila, E., Peña, S., Wismar, M., & Cook, S. (2013). *Health in All Policies*.

Lloret, J., Rätz, H.-J., Lleonart, J., Demestre, M., 2016. Challenging the links between seafood and human health in the context of global change. J. Mar. Biolog. Assoc. U.K., 96(01), 29–42. http://doi.org/10.1017/S0025315415001988

Lucrezi, S., Milanese, M., Markantonatou, V., Cerrano, C., Sarà, A., Palma, M., Saayman, M., 2017. Scuba diving tourism systems and sustainability: Perceptions by the scuba diving industry in two Marine Protected Areas. Tour. Manag., 59, 385–403. http://doi.org/10.1016/J.TOURMAN.2016.09.004

Maragkogianni, A., and Papaefthimiou, S., 2015. Evaluating the social cost of cruise ships air emissions in major ports of Greece. Transp. Res. D. Transp. Environ., *36*, 10–17. http://doi.org/10.1016/j.trd.2015.02.014

MARINE PROTECTED AREAS – MedPAN. 2020. Retrieved May 25, 2020, from https://medpan.org/marine-protected-areas/

Martin, V. Y., Weiler, B., Reis, A., Dimmock, K., Scherrer, P., 2017. 'Doing the right thing': How social science can help foster pro-environmental behaviour change in marine protected areas. *Mar. Policy*, *81*, 236–246. http://doi.org/10.1016/J.MARPOL.2017.04.001

Murray, C. C., Pakhomov, E. A., Therriault, T. W., 2011. Recreational boating: a large unregulated vector transporting marine invasive species. Divers. Distrib., 1–12. http://doi.org/10.1111/j.1472-4642.2011.00798.x

Peters, F. 2019. Canvi climàtic i salut a Catalunya: informe de la Secció de Ciències Biològiques de l'Institut d'Estudis Catalans. Marrasé, C., Camí, J. (eds.). Institut d'Estudis Catalans, Barcelona, 50 pp.

Prata, J. C., da Costa, J. P., Lopes, I., Duarte, A. C., Rocha-Santos, T., 2020. Environmental exposure to microplastics: An overview on possible human health effects. Sci. Total Environ. Elsevier B.V. http://doi.org/10.1016/j.scitotenv.2019.134455

Rako, N., Fortuna, C. M., Holcer, D. Š., Mackelworth, P., Nimak-Wood, M., Pleslić, G., Picciulin, M., 2013. Leisure boating noise as a trigger for the displacement of the bottlenose dolphins of the Cres-LoŠinj archipelago (northern Adriatic Sea, Croatia). *Mar. Pollut. Bull.*, *68*(1–2), 77–84. http://doi.org/10.1016/j.marpolbul.2012.12.019

Rees, S. E., Mangi, S. C., Hattam, C., Gall, S. C., Rodwell, L. D., Peckett, F. J., Attrill, M. J., 2015. The socio-economic effects of a Marine Protected Area on the ecosystem service of leisure and recreation. Mar. Policy, *62*, 144–152. http://doi.org/10.1016/J.MARPOL.2015.09.011

Román, G. C., Jackson, R. E., Gadhia, R., Román, A. N., Reis, J., 2019. Mediterranean diet: The role of long-chain ω-3 fatty acids in fish; polyphenols in fruits, vegetables, cereals, coffee, tea, cacao and wine; probiotics and vitamins in prevention of stroke, age-related cognitive decline, and Alzheimer disease. Rev. Neurol. Elsevier Masson SAS. http://doi.org/10.1016/j.neurol.2019.08.005

Smith, M., Love, D. C., Rochman, C. M., Neff, R. A., 2018. Microplastics in Seafood and the Implications for Human Health. *Curr. Environ. Health Rep.*, *5*(3), 375–386. http://doi.org/10.1007/s40572-018-0206-z

Trabalón, L., Vilavert, L., Domingo, J.L., Pocurull, E., Borrull, F., Nadal, M., 2017. Human exposure to brominated flame retardants through the consumption of fish and shellfish in Tarragona County (Catalonia, Spain), Food Chem. Toxicol., 104, 2017, Pages 48-56.

Trainer, V. L., and Hardy, F. J., 2015. Integrative monitoring of marine and freshwater harmful algae in washington state for public health protection. *Toxins*, *7*(4), 1206–1234. http://doi.org/10.3390/toxins7041206

Valavanidis, A., 2018. Environmental Pollution of Marine and Costal Areas in Greece. Review on marine pollution, monitoring and quality of seawater.

Venturini, S., Massa, F., Castellano, M., Fanciulli, G., Povero, P., 2018. Recreational boating in the Portofino Marine Protected Area (MPA), Italy: Characterization and analysis in the last decade (2006–2016) and some considerations on management. Mar. Policy. http://doi.org/10.1016/J.MARPOL.2018.06.006

Venturini, S., Massa, F., Castellano, M., Costa, S., Lavarello, I., Olivari, E., Povero, P., 2016. Recreational Boating in Ligurian Marine Protected Areas (Italy): A Quantitative Evaluation for a Sustainable Management. Environ. Manage., 57(1), 163–175. http://doi.org/10.1007/s00267-015-0593-y

Verdura, J., Linares, C., Ballesteros, E., Coma, R., Uriz, M. J., Bensoussan, N., Cebrian, E., 2019. Biodiversity loss in a Mediterranean ecosystem due to an extreme warming event unveils the role of an engineering gorgonian species. Nature Sci. Rep., *9*(5911). http://doi.org/10.1038/s41598-019-41929-0

Vila, M., R., Abós-Herràndiz, J., Isern-Fontanet, J., Àlvarez, E. Berdalet, E., 2016. Establishing the link between Ostreopsis cf. ovata blooms and human health impacts using ecology and epidemiology. Sci. Mar. 80S1: 107-115. doi: http://dx.doi.org/10.3989/scimar.04395.08A

Vilavert, L., Borrell, F., Nadal, M., Jacobs, S., Minnens, F., Verbeke, W., Marques, A., Domingo, J.L., 2017. Health risk/benefit information for consumers of fish and shellfish: FishChoice, a new online tool. Food Chem. Toxicol., 104, 79-84.

Wells, S., Ray, G. C., Gjerde, K. M., White, A. T., Muthiga, N., Bezaury Creel, J. E., Causey, Billy D., McCormick-Ray, J., Salm, R., Gubbay, S., Kelleher, G., Reti, J. (2016). Building the future of MPAs – lessons from history. *Aquat. Conserv.*, *26*, 101–125. http://doi.org/10.1002/aqc.2680

Table S4. List of major human health issues and associated goals within the framework of OHH proposed by the Chair's stakeholders. Key references used are shown.

Health issue	Goal	Key references
Sociocultural and economic factors related to health (health inequalities).	Consider the equal and fair access to safe, nutritious, and sustainable seafood by different collectives and ethnic groups.<br>Consider the salutogenic effects of leisure activities and coastal proximity among socioeconomically deprived communities since these effects could be stronger among these populations.	Leppo et al., 2013;<br>Miller et al., 2012
Community health promotion and engagement.	Improve health and wellbeing outcomes by promoting a healthy, active life on the coast or in the sea through the design of "Blue Prescriptions" (similar to the so-called "Green Prescriptions"), as part of patients' health management in primary care centers.<br>Embrace the "asset approach" and the "salutogenic model", which value the capacity, skills, knowledge, connections, and potential in a community (with particular emphasis in empowering women in community health)	Improvement and Development Agency (Great Britain), 2010;<br>Lindström et al., 2005
Blue Spaces as a tool for the improvement of the individual and collective wellbeing of particular vulnerable and at-risk populations.	Propose blue spaces as sites where vulnerable and at-risk populations such as migrants and refugee communities, patients recovering from clinical treatments, and the elderly can carry out healthy activities	Departament de Salut, Generalitat de Catalunya, 2014;<br>Gascon et al., 2017;<br>Vert et al., 2019
Historic wisdom: traditional diet and therapies.	Rediscover the importance of maritime leisure activities to prevent and/or restore health by learning from history regarding the use of the coast and the seas.<br>Rediscover the importance of seafood as a component part of the so-called "Mediterranean diet", including the promotion of exploited, sustainable species that have fallen into disuse and that may contribute to a balanced diet<br>Better understand of the evolution of coastal communities towards medicalization in the 20th century.	Mentella et al., 2019;<br>Schwingshackl et al., 2017
Blue zones	Understand why people live longer (areas with a high percentage of centenarians) and enjoy a better quality of life (so-called Blue Zones) in certain areas and communities located on the coast.	Buettner et al., 2016
Prevention of non-communicable diseases (NCDs)	Promote seafood in the diet and/or physical activity on the coast and at sea to help prevent diseases such as cardiovascular diseases, cancer, and mental disorders.	https://www.seafish.org/promoting-seafood

		Wurzbach, 2002
New medicines from the sea for the treatment of rare diseases and pain	Focus on the bioactive potential of certain marine organisms that can offer unique resources for the discovery of new treatments for rare diseases (e.g., the brevetoxins produced by some dinoflagellate species as a new therapy for cystic fibrosis, and the tetrodotoxins produced by some pufferfish species as a therapeutic agent for pain).	Bhatia et al., 2015; Nieto et al., 2012; Potera, 2007; Sharma et al., 2010

References

Bhatia, P., & Chugh, A. (2015). Role of marine bioprospecting contracts in developing access and benefit sharing mechanism for marine traditional knowledge holders in the pharmaceutical industry. *Global Ecology and Conservation*, *3*, 176–187. http://doi.org/10.1016/j.gecco.2014.11.015

Buettner, D., & Skemp, S. (2016, September 1). Blue Zones: Lessons From the World's Longest Lived. *American Journal of Lifestyle Medicine*. SAGE Publications Inc. http://doi.org/10.1177/1559827616637066

Departament de Salut, Generalitat de Catalunya (2014) Bases per a la promoció de l'envelliment actiu i saludable.

Gascon, M., Zijlema, W., Vert, C., White, M. P., & Nieuwenhuijsen, M. J. (2017). Outdoor blue spaces, human health and well-being: A systematic review of quantitative studies. *International Journal of Hygiene and Environmental Health*, *220*(8), 1207–1221. http://doi.org/10.1016/j.ijheh.2017.08.004

Improvement and Development Agency (Great Britain). (2010). *A glass half-full : how an asset approach can improve community health and well-being*. IDeA.

Leppo, K., Ollila, E., Peña, S., Wismar, M., & Cook, S. (2013). *Health in All Policies*.

Lindström, B., & Eriksson, M. (2005, June). Salutogenesis. *Journal of Epidemiology and Community Health*. J Epidemiol Community Health. http://doi.org/10.1136/jech.2005.034777

Mentella, M. C., Scaldaferri, F., Ricci, C., Gasbarrini, A., & Miggiano, G. A. D. (2019, September 1). Cancer and mediterranean diet: A review. *Nutrients*. MDPI AG. http://doi.org/10.3390/nu11092059

Miller, D., Roe, J., Brown, C., Morris, S., Morrice, J. and Ward Thompson, C. (2012) Blue Health: Water, health and wellbeing, Centre of Expertise for Waters, James Hutton Institute, Aberdeen. Available online at: www.crew.ac.uk/publications

Nieto, F. R., Cobos, E. J., Tejada, M. Á., Sánchez-Fernández, C., González-Cano, R., & Cendán, C. M. (2012, February). Tetrodotoxin (TTX) as a therapeutic agent for pain. *Marine Drugs*. Mar Drugs. http://doi.org/10.3390/md10020281

Potera, C. (2007, June 15). Florida red tide brews up drug lead for cystic fibrosis. *Science*. NIH Public Access. http://doi.org/10.1126/science.316.5831.1561

Schwingshackl, L., Schwedhelm, C., Galbete, C., & Hoffmann, G. (2017, September 26). Adherence to mediterranean diet and risk of cancer: An updated systematic review and meta-analysis. *Nutrients*. MDPI AG. http://doi.org/10.3390/nu9101063

Sharma, M., Sasvari, Z., & Nagy, P. D. (2010). Inhibition of Sterol Biosynthesis Reduces Tombusvirus Replication in Yeast and Plants. *Journal of Virology*, *84*(5), 2270–2281. http://doi.org/10.1128/jvi.02003-09

Vert, C., Nieuwenhuijsen, M., Gascon, M., Grellier, J., Fleming, L. E., White, M. P., & Rojas-Rueda, D. (2019). Health benefits of physical activity related to an urban riverside regeneration. *International Journal of Environmental Research and Public Health*, *16*(3). http://doi.org/10.3390/ijerph16030462

Wurzbach, M. E. (2002). *Community Health Education and Promotion: A Guide to Program Design and Evaluation* (Second). . http://doi.org/http://dx.doi.org/10.1016/S0140-6736(11)60686-7
